# Evaluation of Surgical Site Infection Rates in Traumatic Surgical Fixation and Arthroplasty Performed in Laminar Flow Versus Non-laminar Flow Theatres During the COVID Pandemic

**DOI:** 10.7759/cureus.69154

**Published:** 2024-09-11

**Authors:** Daniel A Lewandowski, Adnan Hussain, Charki Chun, Lynden Chiang, Sashin Ahuja

**Affiliations:** 1 Trauma and Orthopaedics, Univeristy Hospital of Wales, Cardiff, GBR; 2 Trauma and Orthopaedics, University Hospital of Wales, Cardiff, GBR

**Keywords:** covid-19 outbreak, laminar flow, non-laminar flow, surgical site infections, surgical site infections (ssi), trauma & orthopaedics, trauma surgery

## Abstract

Introduction

Laminar flow (LF) in theatres has become the standard of care in orthopaedic implant surgery. Most of the evidence for laminar flow use is based on arthroplasty surgery, with early studies showing a significant reduction in infections. We conducted a retrospective comparative study to assess surgical site infection (SSI) rates in consecutive patients undergoing surgery for trauma in LF and non-laminar flow (NLF) theatres.

Methods

Due to COVID-19 safety restrictions, trauma surgery was performed in non-laminar flow theatres during the pandemic. We identified consecutive patients who had trauma surgery pre- and post-pandemic from February 2019 to June 2021 to avoid selection bias. A total of 1809 patients were identified for the study, and the relevant patient details were collected through the hospital operating theatre software (Bluespier) and patient records (Welsh Clinical Portal). There were 917 in the laminar theatre group and 892 in the non-laminar theatre group. For the purpose of this study, we recorded SSI rates within the first 90 days. The two groups were statistically similar in terms of age and gender of the patients.

Results

Nineteen patients developed surgical site infections in non-laminar flow theatres and 25 patients in laminar flow theatres. There was no significant difference between the SSI rate in laminar flow theatres (2.72%) as compared to non-laminar flow theatres (2.13%) (p=0.399). There was no link between infections and the duration of surgery. Two patients in the laminar flow group were MRSA-positive and were excluded.

Conclusion

In our study, the laminar flow theatres did not show a statistically significant reduction in surgical site infections. We conclude in the practical environment of trauma theatres the theoretical advantage of laminar flow does not translate to an observable reduction of infections.

## Introduction

Prevention of surgical site infections (SSI) is a key priority in orthopaedic implant surgeries. These include primary joint arthroplasties for trauma and open reduction internal fixation using implants. An additional infection risk in orthopaedic implant surgery is the formation of biofilm on the implant surface. It makes these infections harder to eradicate, necessitating secondary surgeries for implant removal in certain cases. Since the development of ultra-clean air operating theatres by Professor Sir John Charnley, the use of laminar flow (LF) theatres, in addition to prophylactic antibiotics, has been recommended and remains the gold standard practice to decrease SSI.

The Lidwell trial [1] showed improved infection rates by using LF theatres but did not account for the benefit of prophylactic antibiotics in that study. Studies have shown LF theatres have lower airborne bacterial counts; however, recent studies and meta-analyses have shown that this may not translate into improving SSI rates [2-4]. Furthermore, a retrospective cohort study on hip and knee arthroplasties conducted in the United States found no association between the two theatres with SSI rates of 0.5% and 0.4%, respectively [2]. A study in the United Kingdom found LF had a p = <0.33 and non-laminar flow (NLF) a p = <0.59 for SSI rates in hip and knee arthroplasties, not statistically different enough (p = <0.39) for a solid conclusion [3]. A London study found SSI rates lower in NLF theatres (2.2%) vs. LF (4.7%) [4]. A meta-analysis of eight cohort study papers found no increased risk for SSI rates following NLF hip and knee arthroplasties, and a German study recorded increased SSIs for hip and knee prosthesis surgeries in LF (1.85% and 1.33%) compared to NLF theatres (1.31% and 0.82%) [5,6].

In our units, all orthopaedic implant surgeries were routinely performed under laminar flow theatres, but during the COVID-19 pandemic, we moved to NLF theatres due to local policies. We have evaluated the surgical site infection rates that result from that change in our study, which focusses on trauma patients. This article was previously presented as an oral presentation at the 2023 Association of Surgeons in Training Conference and at the 2023 Welsh Orthopaedics Society Meeting. Most previous studies have concentrated on arthroplasty surgery, and not many studies evaluate general trauma patients on this subject.

## Materials and methods

A total of 1809 consecutive patients who had trauma surgery were identified from February 19, 2019, to June 14, 2021. All trauma patients needing any form of fixation in terms of open reduction internal fixations, joint hemiarthroplasties, or total joint replacements were included in the study. Elective operating lists were cancelled during the pandemic, so elective patients were excluded. The study included all patients requiring surgery; from February 2019 to March 2020, we operated on 917 patients in a laminar flow theatre, and from March 2020 to June 2021, we operated on 892 patients in a non-laminar flow theatre, following changes in guidelines due to the COVID pandemic, across two operating sites: University Hospital of Wales (UHW) and University Hospital of Llandough (UHL).

**Figure 1:**
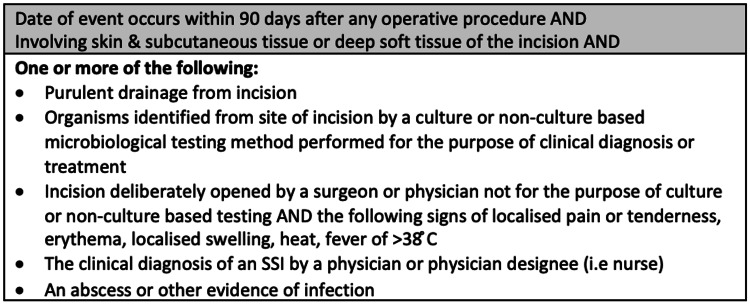
CDC surgical site infection criteria Image Credit: Daniel Lewandowski. Information taken from CDC Website [7].

The relevant patient details were collected through the hospital operating theatre software Bluespier and patient records on the Welsh Clinical Portal. Bluespier includes details of the surgical operation, the American Society of Anaesthesiologists (ASA) score, and necessary pre-operative patient notes, which were recorded. Not all patients had ASA scores in their surgical notes, so this was not included in the report. Bluespier was used to obtain patients who underwent NLF orthopaedic trauma replacement surgeries at both UHW and UHL between March 2020 and February 2021. WCP was then cross-referenced to collate individual patient’s pre- and post-operative notes to further analyse their discharge summaries, clinical follow-ups, and investigations. Further, the program mainly focused on increased white cell counts, C-reactive protein levels, microbiology results, or wound swabs to indicate a potential post-operative SSI infection, which was then further investigated using the CDC SSI guidelines (Figure 1) [7]. This process was repeated for laminar flow orthopaedic trauma replacement surgery patients at University Hospital Wales between March 2019 and December 2019. The PICO model (Patient, Intervention, Comparison, and Outcome) was applied, a technique used to formulate clinical questions covering patient information, exposure to treatment, and outcomes (P: patients undergoing orthopaedic trauma replacement surgery; I: surgical replacements in laminar flow theatres; C: surgical replacements in non-laminar flow theatres; O: post-operative SSI as per CDC guidance) [8]. Two sample t-tests were used, and the value for Ho was taken from the Lidwell paper, assuming 0.6% will have SSIs in LF and 1.5% will have SSIs in NLF [1]. A T-test was used for continuous variables, and a Chi-square test or Fisher exact test was used for categorical variables; a p-value less than (<) 0.05 is significant, otherwise non-significant. These were all obtained using Statistical Package for the Social Sciences (SPSS, IBM Corp., Armonk, NY) software.

As per CDC guidelines for replacement surgeries, an SSI occurs within 90 days of the event; thus, infections occurring past this were excluded [7]. Patients who had revision surgery due to a previous post-operative SSI or patients presenting with cellulitis post-operatively without specific identification of an SSI were not included in the results.

## Results

The provided tables compare demographic data, SSIs, and HAIs between patients undergoing surgeries in LF and NLF operating theatres across various surgical procedures. Of the 1809 trauma patients identified between February 2019 and June 2021, 917 patients had operations in an LF theatre and 892 in an NLF theatre. Of the 892 operated in NLF theatres, 543 were operated at our emergency site with direct accident and emergency admissions at UHW and 349 at our elective site with no direct admissions at UHL (Table 1). Operations were analyzed by joint hemiarthroplasty (Table 2), total joint arthroplasty (Table 3), and open reduction internal fixation procedures (Table 4).

**Table 1:** Demographics and SSIs in all patients in laminar and non-laminar flow Independent T-tests were used for continuous variables, and chi-square tests or Fisher exact tests were used for categorical variables, using SPSS software. A P-value <0.05 is statistically significant. SSI: surgical site infection.

	Laminar flow	Non-laminar flow	Test statistics	P-value
Total patient	917	892		
Sex (M:F)	408:509	367:525	1.937	0.164
Mean age (range in years)	56.2 (1.2–103)	61.2 (0.5–100)	−3.95	<0.001
Mean length of admission (days)	18.6	16.2	1.98	0.047
Risk factor (Y/N)	215:702	296:596	20.677	<0.001
Comorbidities (Y/N)	521:396	540:352	2.4323	0.1189
Procedure				
Hemiarthroplasty SSI	3	1	3.1885	0.2031
Arthroplasty SSI	0	2		
Fixation SSI	22	16		
Total infection				
Total surgical site infections	25	19	0.70863	0.3999
Hospital-acquired infections	86	92		

**Table 2:** Joint hemiarthroplasty in laminar flow versus non-laminar flow theatres Independent T-tests were used for continuous variables, and chi-square tests or Fisher exact tests were used for categorical variables, using SPSS software. P-value <0.05 is statistically significant.

	Laminar flow	Non-laminar flow	Test statistics	P-value
Total patient	154	160		
Sex (M:F)	46:108	50:110	0.02039	0.8864
Mean age (range in years)	82.6 (46–103)	81.9 (54–99)	0.605	0.546
Mean length of admission (days)	41.14	24.27	4.41	<0.001
Procedure				
Hip joint procedure	152	154	0.220779	0.2936
Elbow joint procedure	2	3		
Shoulder joint procedure	0	3		
Knee joint procedure	0	0		
Infections				
Total surgical site Infections	3	1	0.26562	0.6063
Hospital acquired Infections	31	33		

**Table 3:** Total joint arthoplasty in laminar versus non-laminar flow theatres An independent T-test was used for continuous variables, and a chi-square test or Fisher exact test was used for categorical variables, using SPSS software. P-value <0.05 is statistically significant.

	Laminar flow	Non-laminar flow	Test statistics	P-value
Total patient	34	98		
Sex (M/F)	13:21	31:67	0.24265	0.6223
Mean age (range in years)	73.44	70.8	0.115	0.326
Mean length of admission (days)	13.4	15.9	0.314	0.123
Procedure				
Hip joint procedure	25	67	1.0497	0.7805
Elbow joint procedure	2	4		
Shoulder joint procedure	2	11		
Knee joint procedure	5	16		
Infections				
Total surgical site infections	0	2	0.00328	0.9543
Hospital acquired infections	4	9		

The slight difference in patient numbers (917 vs. 892) suggests a comparable distribution of cases between the two environments. The male-to-female ratio was slightly different between the groups (408:509 in LF vs. 367:525 in NLF), but this difference was not statistically significant (p=0.164) (Table 1). The mean age of patients was significantly lower in LF theatres (56.2 years) compared to NLF theatres (61.2 years) with a p-value of <0.001, indicating a statistically significant difference in age distribution. Patients in LF theatres had a longer mean length of admission (18.6 days) compared to NLF theatres (16.2 days), and this difference was statistically significant (p=0.047). The presence of comorbidities was slightly higher in NLF theatres, but this difference was not statistically significant (p=0.1189). The total SSIs were slightly higher in LF theatres (25 vs. 19), but this difference was not statistically significant (p=0.3999). Similarly, HAIs were marginally higher in NLF theatres (92 vs. 86), but this difference was also not significant (Table 1).

**Table 4:** Open reduction and internal fixation (ORIF) procedures in laminar versus non-laminar flow theatres Independent T-tests were used for continuous variables, and the chi-square test or Fisher exact test was used for categorical variables, using SPSS software. P-value <0.05 is statistically significant.

	Laminar Flow	Non-Laminar Flow	Test statistics	P-value
Total patient	729	634		
Sex (M/F)	349:380	288:346	0.7209	0.3958
Mean age (range in years)	49.7	54.59	0.305	0.146
Mean length of admission (days)	15.48	13.57	0.431	0.201
Procedure				
Hip joint procedure	249	235	20.308	0.000147
Upper limb procedure	236	184		
Lower limb procedure	230	175		
Spinal procedure	14	40		
Infections				
Total surgical site infections	22	16	0.34586	0.5565
Hospital acquired infections	51	50		

Regarding joint hemiarthroplasties, LF theatres had 154 patients compared to 160 in NLF theatres (Table 2). The mean age was similar between the two groups, with no significant difference (p=0.546). The male-to-female ratio also showed no significant variation (p=0.8864). A significant difference was observed, with a longer mean length of admission in LF theatres (41.14 days vs. 24.27 days; p<0.001). SSIs were slightly more frequent in LF theatres (3 vs. 1), but this was not statistically significant (p=0.6063). The distribution of HAIs was almost equal between the two groups.

In total joint arthroplasties, this category had fewer patients overall, with 34 in LF theatres and 98 in NLF theatres (Table 3). The mean age and sex distribution did not show significant differences (p=0.326 for age and p=0.6223 for sex). Patients in NLF theatres had a longer mean length of admission (15.9 days) compared to those in LF theatres (13.4 days), although this difference was not statistically significant (p=0.123). Notably, no SSIs were reported in LF theatres, while two were reported in NLF theatres. However, this difference was not statistically significant (p=0.9543).

The biggest group of patients fell under open reduction internal fixation (ORIF). LF theatres saw 729 patients, whereas NLF theatres treated 634 (Table 4). The differences in mean age (p=0.146) and sex distribution (p=0.3958) were not statistically significant. The mean length of admission was longer in LF theatres (15.48 days vs. 13.57 days), though this was not significant (p=0.201). SSIs were slightly more common in LF theatres (22 vs. 16), but the difference was not statistically significant (p=0.5565).

An analysis of NLF site 1 infection post-operatively demonstrated soft tissue infections 15/543 (2.76%) and hospital-acquired infections 71/543 (13.07%), whilst NLF site 2 infections post-operatively resulted in 4/349 (1.15%) due to soft tissue and 21/349 (6.02%) secondary to hospital-acquired infections (Table 1). A look at LF patients showed 25/917 (2.72%) with soft tissue infections and 86/917 (9.37%) hospital-acquired infections (Table 1). SSI infection rates were calculated to be 12.44% in NLF theatres and 12.1% in LF theatres and proved to have no statistical difference with a calculated p-value of 0.3999. The findings conclude that trauma patients have no significant advantage in using laminar flow theatres when compared to non-laminar flow theatres.

## Discussion

SSIs can result in serious postoperative complications that significantly delay a patient's recovery and, in the most severe cases, may even be life-threatening. Some studies have demonstrated LF ventilation is more effective in reducing SSI rates compared to conventional mixing ventilation [9,10]. Our study shows no difference in SSI rates when LF and NLF theatre use is compared, and several papers also display a similar pattern to our results [3,11]. A systematic review by Gastmeier et al. actually found there to be increased SSI rates with LF use [12]. Despite these inconsistencies, LAF is still widely considered the optimal form of OR ventilation according to many national standards.

One possible explanation for the lack of a significant difference in SSI rates between LF and NLF use in theatres could be the role of other infection control measures, many of which have become standard practice in today’s surgical care. For example, the widespread use of prophylactic antibiotics has been shown to significantly reduce SSIs [13], potentially diminishing the relative impact of LF systems. Furthermore, proper hand hygiene, use of sterile barriers, careful wound closure, and other infection prevention practices may also contribute to infection prevention [14]. 

Another factor to consider is the varied effectiveness of LF systems due to real-world operating conditions. Studies have highlighted that LF systems are sensitive to disruptions caused by operating staff movements, placement of surgical lights, and patient warming devices, all of which can create turbulence and disturb the intended airflow patterns [5,6]. Such disruptions can lead to localized increases in bacterial counts near the surgical site, negating the theoretical advantages of LF ventilation.

Furthermore, it may be the case that once a decontamination threshold is achieved, further reduction in bacterial count may not contribute to further improvements in clinical infection rates. A recent experimental study showed that bacterial counts significantly vary in different parts of the theatre and are influenced by many structures present, which may result in higher bacterial counts over the wound sites [15].

Although it may be true that LF theatres have been shown to decrease airborne pathogen counts under controlled conditions, in the day-to-day environment of trauma theatres, these conditions are not met, and often, the theoretical advantage of laminar flow does not translate to a direct advantage of reduction of infections [2,7,16]. Overall, the implementation of a perfect laminar flow system necessitates the control of numerous variable factors, which may not be feasible in a real-world healthcare environment. Consequently, recent research does not definitively support this ventilation method.

## Conclusions

This study does not show any benefit of using LF versus NLF theatres for trauma patients, including for arthroplasties. Infection rates are influenced by many factors. Good surgical discipline, adequate maintenance of sterile equipment, and the use of appropriate prophylactic antibiotics all play a significant role in optimizing patient safety and decreasing SSIs. These findings align with recent literature questioning the clinical efficacy of LF systems in reducing infection rates. Factors such as operating room temperature, turbulence caused by patient warming, theatre lights, and staff movements may all interfere with the effectiveness of LF systems, potentially diminishing their benefits.

Given the lack of a significant difference in infection rates from this study, routine use of LF theatres for trauma surgeries may not be necessary. It remains crucial to emphasize established infection control measures, including strict adherence to aseptic techniques, thorough sterilization of instruments, and careful use of antibiotics. Future studies with larger sample sizes and more controlled environments could clarify the specific circumstances in which LF theatres may provide clinical advantages.
